# Supplementary material for: Comparison of species-specific qPCR and metabarcoding methods to detect small pelagic fish distribution from open ocean environmental DNA
Source: PLoS One. 2022 Sep 7;17(9):e0273670. doi: 10.1371/journal.pone.0273670 (PMC9451083; doi:10.1371/journal.pone.0273670)
Supplement: S1 Text — (PDF) [file pone.0273670.s001.pdf]

S1 Text. Detailed procedure for the MiFish method.

1. Library preparation and sequencing method

1). DNA extraction

DNA extraction was performed using ChargeSwitch Forensic DNA Purification Kit (Thermo Fisher Scientific). However, when tried to use the eluted DNA solutions directly for library preparation, the amplification cannot be confirmed in the first-round PCR. Thus, a purification step by adding an equal volume of AMPure XP Reagent (Beckman Coulter Inc., Brea, CA, USA) was performed before quantitative measurement 2).

2). Quantitative measurement of DNA solution

The concentration of the DNA solution was measured using Synergy H1 (Bio Tek) and QuantiFluor dsDNA System (Promega).

3). Library preparation

In library preparation, firstly the 1st PCR was performed with 1st-MiFish primers, then the PCR products were purified, went through the 2nd PCR and purified again to get the library for sequencing. The primers and reaction settings were shown in S1 Fig.

As shown in S1 Fig, the concentration of Template DNA in the 1st PCR was controlled to no more than 2.0 ng/μL, which means DNA samples with higher concentration were diluted to 2.0 ng/μL. For samples of C-line, the library preparation failed in 35 of 130 samples. For the failed samples, the original samples were diluted 10 times (which made their concentration no more than 0.2 ng/μL) to further suppress the possible influence of PCR inhibitor. The second-time library preparation, in which the settings were the same as shown in S1 Fig except the concentration of Template DNA, was performed to those samples.

4). Quantification of library

The concentration of the library was measured using Synergy H1 and QuantiFluor dsDNA System.

5). Library quality check

We confirmed the quality of library created using Fragment Analyzer and dsDNA 915 Reagent Kit (Advanced Analytical Technologies).

6). Sequencing analysis

Sequencing analysis was performed using MiSeq 2x300 bp method to do the Sequencing analysis.

2. Data analysis method

1). Read quality filtering

Read quality filtering was performed using (fastq_barcode_splitter.pl) of Fastx toolkit, only the sequences whose sequence reading start exactly matched the primer used were extracted. The primer sequence and the latter 70 bases of the read were deleted from the extracted sequence. Then, sequences with a quality value of less than 20 were removed, using sickle tools, and sequences with a length of 40 bases or less were discarded together with the paired sequences.

2). Reads merging

We used the pair-end merge script FLASH to merge the arrays through quality filtering.

The conditions for merging were a fragment length of 180 bases after merging, a read fragment length of 170 bases, and a minimum overlap length of 10 bases.

3). OTU preparation and identification

OTU creation and identification was performed using USEARCH under conditions of 97% sequence homology. The created OTU was phylogenetically estimated by comparing it with the sequence of the fish mitochondrial genome database (MitoFish) and the reference for MiFish using BLAST search. To avoid false positives, only when an OTU has reads higher than 1% reads sum of one sample, it was admitted as a positive OTU in this sample.
