# Supplementary material for: Comparison of species-specific qPCR and metabarcoding methods to detect small pelagic fish distribution from open ocean environmental DNA
Source: PLoS One. 2022 Sep 7;17(9):e0273670. doi: 10.1371/journal.pone.0273670 (PMC9451083; doi:10.1371/journal.pone.0273670)
Supplement: S1 Fig — (PDF) [file pone.0273670.s002.pdf]

S1 Fig. The library procedure, thermal cycle settings, and primer sequences of the MiFish method in this study.

Procedure of library preparation

1st PCR(repeat 4 times)

↓

DNA purification

↓

2nd PCR

↓

DNA purification

↓

Library preparation finished

1st PCR(repeat 4 times)
reaction system:

10×Ex Buffer	1.0μl
dNTPs(each 2.5mM)	0.8μl
10μM Forward primer	0.5μl
10μM Reverse primer	0.5μl
Template DNA(max 2ng/μl)	2.0μl
ExTaq[TaKaRa](5U/μl)	0.1μl
Double distilled water	5.1μl
Total	10.0μl

Thermal cycles setting:

95°C	3min	
98°C	20s	35 cycles
65°C	15s	35 cycles
72°C	20s	35 cycles
72°C	5min	

Primer sequence:

primer name	Sequence(5’→3’)
1st-MiFish U-F	ACACTCTTTCCCTACACGACGCTCTTCCGATCTNNNNNNGTCGGTAAAACTCGTGCCAGC
1st-MiFish E-F	ACACTCTTTCCCTACACGACGCTCTTCCGATCTNNNNNNGTTGGTAAATCTCGTGCCAGC
1st-MiFish U-R	GTGACTGGAGTTCAGACGTGTGCTCTTCCGATCTNNNNNNCATAGTGGGGTATCTAATCCCAGTTTG
1st-MiFish E-R	GTGACTGGAGTTCAGACGTGTGCTCTTCCGATCTNNNNNNCATAGTGGGGTATCTAATCCCAGTTTG

2nd PCR
reaction system:

10×Ex Buffer	1.0μl
dNTPs(each 2.5mM)	0.8μl
10μM Forward primer	0.5μl
10μM Reverse primer	0.5μl
products from 1st PCR(max 2ng/μl)	2.0μl
ExTaq[TaKaRa](5U/μl)	0.1μl
Double distilled water	5.1μl
Total	10.0μl

Thermal cycles setting:

94°C	2min	
94°C	30s	12 cycles
60°C	30s	12 cycles
72°C	30s	12 cycles
72°C	5min	

Primer sequence:

primer name	Sequence(5’→3’)
2nd F	AATGATACGGCGACCACCGAGATCTACAC-Index2*-ACACTCTTTCCCTACACGACGC
2nd R	CAAGCAGAAGACGGCATACGAGAT-Index1*-GTGACTGGAGTTCAGACGTGTG

*Recorded in S3 Table.
