# Supplementary material for: Comparison of species-specific qPCR and metabarcoding methods to detect small pelagic fish distribution from open ocean environmental DNA
Source: PLoS One. 2022 Sep 7;17(9):e0273670. doi: 10.1371/journal.pone.0273670 (PMC9451083; doi:10.1371/journal.pone.0273670)
Supplement: S2 Fig — (PDF) [file pone.0273670.s003.pdf]

S2 Fig.

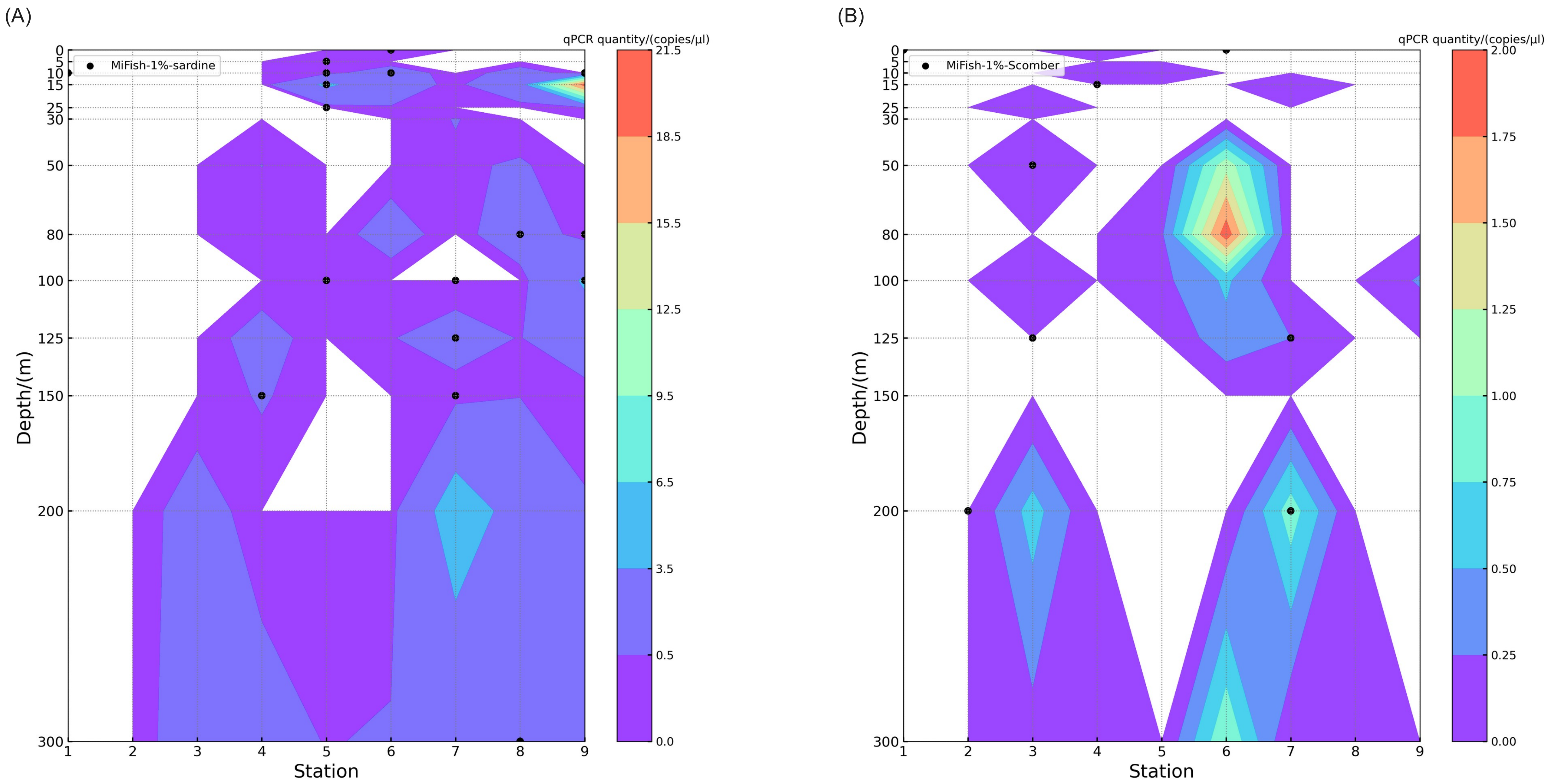
(A)
qPCR quantity/(copies/μl)
MiFish-1%-sardine
Depth/(m)
Station
0
5
10
15
25
30
50
80
100
125
150
200
300
1
2
3
4
5
6
7
8
9
21.5
18.5
15.5
12.5
9.5
6.5
3.5
0.5
0.0
(B)
qPCR quantity/(copies/μl)
MiFish-1%-Scomber
Depth/(m)
Station
2.00
1.75
1.50
1.25
1.00
0.75
0.50
0.25
0.00

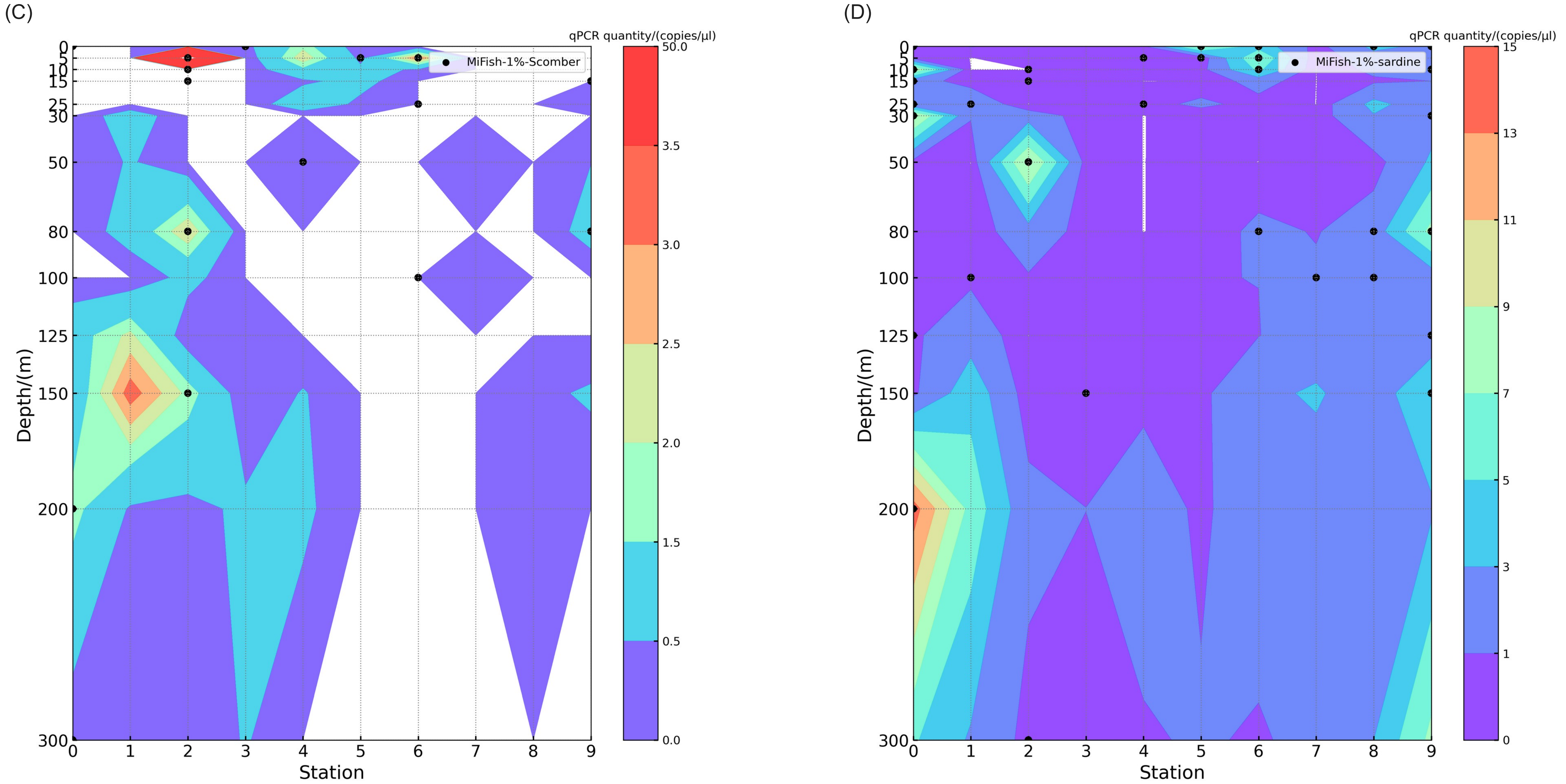

S2 Fig. Comparison between the spatial distribution of presence detection samples found by MiFish and the eDNA quantity obtained from the qPCR results.

A) Results of chub/blue mackerel(*Scomber japonicus* and *Scomber australasicus*), and B) results of sardine(*Sardinops melanostictus*) in the B-line vertical section, C) results of chub/blue mackerel(*Scomber japonicus* and *Scomber australasicus*) and D) results of sardine(*Sardinops melanostictus*) in the C-line vertical section. Filled circles represent the presence detection samples found by the MiFish method. Background colors represent the eDNA quantity from the qPCR method.
