# Supplementary material for: Comparison of species-specific qPCR and metabarcoding methods to detect small pelagic fish distribution from open ocean environmental DNA
Source: PLoS One. 2022 Sep 7;17(9):e0273670. doi: 10.1371/journal.pone.0273670 (PMC9451083; doi:10.1371/journal.pone.0273670)
Supplement: S8 Table — (PDF) [file pone.0273670.s011.pdf]

S8 Table. Comparison tables for Phi-coefficients analysis of detection performance. 0 = no detection, 1 = positive detection, before and after omitting samples that library preparation was failed.

original (247 samples)

Scomber australasicus and *Scomber japonicus*

		MiFish 1	MiFish 0
qPCR	1	12	51
	0	11	173

Engraulis japonicus

		MiFish 1	MiFish 0
qPCR	1	1	15
	0	3	228

Sardinops melanostictus

		MiFish 1	MiFish 0
qPCR	1	43	111
	0	6	87

Cololabis saira

		MiFish 1	MiFish 0
qPCR	1	1	1
	0	6	239

Trachurus japonicus

		MiFish 1	MiFish 0
qPCR	1	0	1
	0	0	246

after omitting (187 samples)

Scomber australasicus and *Scomber japonicus*

		MiFish 1	MiFish 0
qPCR	1	12	45
	0	11	116

Engraulis japonicus

		MiFish 1	MiFish 0
qPCR	1	1	13
	0	3	167

Sardinops melanostictus

		MiFish 1	MiFish 0
qPCR	1	43	88
	0	6	47

Cololabis saira

		MiFish 1	MiFish 0
qPCR	1	1	0
	0	6	177

Trachurus japonicus

		MiFish 1	MiFish 0
qPCR	1	0	1
	0	0	186
