# Supplementary material for: Comparison of species-specific qPCR and metabarcoding methods to detect small pelagic fish distribution from open ocean environmental DNA
Source: PLoS One. 2022 Sep 7;17(9):e0273670. doi: 10.1371/journal.pone.0273670 (PMC9451083; doi:10.1371/journal.pone.0273670)
Supplement: Attachment — Submitted filename: Response_to_Reviewers_YUzeshu_20220429.docx [file pone.0273670.s013.docx]

**Response to academic editor and reviewers**

We would like to thank the academic editor and reviewers for their encouragement and their devoted efforts to review the manuscript. We have done our best to address the concerns below. We much appreciate the reviewers for your thorough and constructive reviews, which we think have greatly improved the manuscript. Our responses are in blue and red (red color marked what we modified in the manuscript), while the original comments are in black and italicized.

Detailed response to academic editor

*1. Please ensure that your manuscript meets PLOS ONE's style*

*requirements, including those for file naming. The PLOS ONE style*

*templates can be found at*

*https://journals.plos.org/plosone/s/file?id=wjVg/PLOSOne_formatting_sample_main_body.pdf*

*<https://journals.plos.org/plosone/s/file?id=wjVg/PLOSOne_formatting_sample_main_body.pdf>*

*and*

*https://journals.plos.org/plosone/s/file?id=ba62/PLOSOne_formatting_sample_title_authors_affiliations.pdf*

*<https://journals.plos.org/plosone/s/file?id=ba62/PLOSOne_formatting_sample_title_authors_affiliations.pdf>*

> Thank you very much for your reminding. This time we carefully checked to make the manuscript meets PLOS ONE's style requirements.

*2. Thank you for stating the following financial disclosure:*

*“SI, JP21H04735, The Japan Society for the Promotion of Science (JSPS)*

*KAKENHI, https://www.jsps.go.jp/english/, NO*

*SI & SH, The OceanDNA project, The University of Tokyo Future Society*

*Initiative, https://www.u-tokyo.ac.jp/adm/fsi/ja/projects/sdgs/projects_00103.html, NO”*

*Please state what role the funders took in the study. If the funders had no role, please state: "The funders had no role in study design, data collection and analysis, decision to publish, or preparation of the manuscript."*

*If this statement is not correct you must amend it as needed.*

*Please include this amended Role of Funder statement in your cover letter; we will change the online submission form on your behalf.*

> Thank you for your reminding. Yes, as you said, the funders had no role in the study, this time we included the statement in the Financial Disclosure section as:

“SI, JP21H04735, The Japan Society for the Promotion of Science (JSPS) KAKENHI, https://www.jsps.go.jp/english/. The funders had no role in study design, data collection and analysis, decision to publish, or preparation of the manuscript.

SI & SH, The OceanDNA project, The University of Tokyo Future Society Initiative,

https://www.u-tokyo.ac.jp/adm/fsi/ja/projects/sdgs/projects_00103.html. The funders had no role in study design, data collection and analysis, decision to publish, or preparation of the manuscript.”

*3. Thank you for stating the following in the Acknowledgments Section of*

*your manuscript:*

*“The contribution of SI was supported by the Japan Society for the Promotion of Science (JSPS) KAKENHI (Grant Number JP21H04735) and the OceanDNA project under the University of Tokyo Future Society Initiative. The OceanDNA survey was conducted by the R/V Shinsei-Maru.*

*We thank the captain and all members of cruise KS-18-5. We also appreciate the assistance of Dr. Megumi Enomoto for sea water sampling.”*

*We note that you have provided additional information within the Acknowledgements Section that is not currently declared in your Funding Statement. Please note that funding information should not appear in the Acknowledgments section or other areas of your manuscript. We will only*

*publish funding information present in the Funding Statement section of*

*the online submission form.*

*Please remove any funding-related text from the manuscript and let us know how you would like to update your Funding Statement. Currently, your* *Funding Statement reads as follows:*

*“SI, JP21H04735, The Japan Society for the Promotion of Science (JSPS)*

*KAKENHI, https://www.jsps.go.jp/english/, NO*

*SI & SH, The OceanDNA project, The University of Tokyo Future Society*

*Initiative, https://www.u-tokyo.ac.jp/adm/fsi/ja/projects/sdgs/projects_00103.html, NO”*

*Please include your amended statements within your cover letter; we will*

*change the online submission form on your behalf.*

> We are sorry for this mistake, this time we deleted the funding-related text in the manuscript and stated the Funding Statement again in the Financial Disclosure section.

*4. We note that figure 1 in your submission contain [map/satellite] images which may be copyrighted. All PLOS content is published under the Creative Commons Attribution License (CC BY 4.0), which means that the manuscript, images, and Supporting Information files will be freely available online, and any third party is permitted to access, download, copy, distribute, and use these materials in any way, even commercially, with proper attribution. For these reasons, we cannot publish previously*

*copyrighted maps or satellite images created using proprietary data, such as Google software (Google Maps, Street View, and Earth). For more information, see our copyright guidelines:*

*http://journals.plos.org/plosone/s/licenses-and-copyright*

*<http://journals.plos.org/plosone/s/licenses-and-copyright>.*

*We require you to either (1) present written permission from the*

*copyright holder to publish these figures specifically under the CC BY*

*4.0 license, or (2) remove the figures from your submission:*

*1. You may seek permission from the original copyright holder of figure 1 to publish the content specifically under the CC BY 4.0 license.*

*We recommend that you contact the original copyright holder with the Content Permission Form*

*(http://journals.plos.org/plosone/s/file?id=7c09/content-permission-form.pdf*

*<http://journals.plos.org/plosone/s/file?id=7c09/content-permission-form.pdf>)*

*and the following text:*

*“I request permission for the open-access journal PLOS ONE to publish XXX under the Creative Commons Attribution License (CCAL) CC BY 4.0*

*(http://creativecommons.org/licenses/by/4.0/*

*<http://creativecommons.org/licenses/by/4.0/>). Please be aware that this license allows unrestricted use and distribution, even commercially, by third parties. Please reply and provide explicit*

*written permission to publish XXX under a CC BY license and complete the attached form.”*

*Please upload the completed Content Permission Form or other proof of granted permissions as an "Other" file with your submission.*

*In the figure caption of the copyrighted figure, please include the following text: “Reprinted from [ref] under a CC BY license, with permission from [name of publisher], original copyright [original*

*copyright year].”*

*2. If you are unable to obtain permission from the original copyright holder to publish these figures under the CC BY 4.0 license or if the copyright holder’s requirements are incompatible with the CC BY 4.0 license, please either i) remove the figure or ii) supply a replacement figure that complies with the CC BY 4.0 license. Please check copyright information on all replacement figures and update the figure caption with source information. If applicable, please specify in the figure caption text when a figure is similar but not identical to the original image and is therefore for illustrative purposes only.*

*The following resources for replacing copyrighted map figures may be*

*helpful:*

*USGS National Map Viewer (public domain):*

*http://viewer.nationalmap.gov/viewer/*

*<http://viewer.nationalmap.gov/viewer/>*

*The Gateway to Astronaut Photography of Earth (public domain):*

*http://eol.jsc.nasa.gov/sseop/clickmap/*

*<http://eol.jsc.nasa.gov/sseop/clickmap/>*

*Maps at the CIA (public domain):*

*https://www.cia.gov/library/publications/the-world- factbook/index.html*

*<https://www.cia.gov/library/publications/the-world-%20factbook/index.html>*

*and*

*https://www.cia.gov/library/publications/cia-maps-publications/index.html <https://www.cia.gov/library/publications/cia-maps-publications/index.html>*

*NASA Earth Observatory (public domain):*

*http://earthobservatory.nasa.gov/ <http://earthobservatory.nasa.gov/>*

*Landsat: http://landsat.visibleearth.nasa.gov/*

*<http://landsat.visibleearth.nasa.gov/>*

*USGS EROS (Earth Resources Observatory and Science (EROS) Center)*

*(public domain): http://eros.usgs.gov/# <http://eros.usgs.gov/>*

*Natural Earth (public domain): http://www.naturalearthdata.com/*

*<http://www.naturalearthdata.com/>*

*[Note: HTML markup is below. Please do not edit.]*

> Thank you very much for your statement about the copyright concerns. When drawing the map in Fig 1, we used the data of Natural Earth. But as stated on the website of Natural Earth (<https://www.naturalearthdata.com/about/terms-of-use/>) as follows, they don’t need users to ask them for permission.

“All versions of Natural Earth raster + vector map data found on this website are in the public domain. You may use the maps in any manner, including modifying the content and design, electronic dissemination, and offset printing. The primary authors, Tom Patterson and Nathaniel Vaughn Kelso, and all other contributors renounce all financial claim to the maps and invites you to use them for personal, educational, and commercial purposes.

No permission is needed to use Natural Earth. Crediting the authors is unnecessary.

However, if you wish to cite the map data, simply use one of the following.

Short text:

Made with Natural Earth.

Long text:

Made with Natural Earth. Free vector and raster map data @ naturalearthdata.com.”

Thus, we added the statement as “Maps were made in Spyder (Python 3.7) using Natural Earth data. Free vector and raster map data @ naturalearthdata.com.” in the caption of Fig 1.

When drawing Fig 1, we also used the current velocity data (Ocean Surface Current Analysis Real-time) and sea surface temperature data (GHRSST Level 4 OSTIA Global Foundation Sea Surface Temperature Analysis) downloaded from the PO.DAAC. But as stated on the website of PO.DAAC (https://podaac.jpl.nasa.gov/CitingPODAAC) as follows, they also don’t need users to ask them for permission.

“Data hosted by the PO.DAAC is openly shared, without restriction, in accordance with NASA's Earth Science program Data and Information Policy.

Bibliographic citations should be included in the References section of publications and other media to acknowledge those who have created the data, services, and tools provided by the PO.DAAC. Proper citations include the authors, title, publisher, and Digital Object Identifier (DOI) and will allow the products to be discovered and re-used by others. The proper citation for each PO.DAAC product is provided on its landing page and user guide.

Example Data Citation

…”

Thus, we modified the statement as “(a) Colors represent current speeds and arrows represent current velocity vectors on the sea surface (5-day mean from May 10 to 14, 2018) from Ocean Surface Current Analysis Real-time data [34] (hosted and openly shared by the PO.DAAC, without restriction, in accordance with NASA's Earth Science program Data and Information Policy). (b) The contours and colors represent sea surface temperature (℃) distribution on May 10, 2018, from GHRSST Level 4 OSTIA Global Foundation Sea Surface Temperature Analysis data [35] (hosted and openly shared by the PO.DAAC, without restriction, in accordance with NASA's Earth Science program Data and Information Policy)” in the caption of Fig 1. And we also added the Citation in the “References” as the follows, like the PO.DAAC website asked for.

“34. ESR. 2009. OSCAR third deg. Ver. 1. PO.DAAC, CA, USA. Dataset accessed [2021-07-08] at https://doi.org/10.5067/OSCAR-03D01.

35. UK Met Office. 2005. OSTIA L4 SST Analysis. Ver. 1.0. PO.DAAC, CA, USA. Dataset accessed [2021-07-06] at https://doi.org/10.5067/GHOST-4FK01.”

And finally, we wish to keep the original Fig 1 in the manuscript with the added citation information showed above.

Detailed response to reviewers

*Reviewer #1:*

*General comments to authors*

*Authors collected large number of eDNA samples from open ocean environment and performed eDNA analysis using both species-specific qPCR and MiFish metabarcoding. The results indicated both methods have good agreement, while the qPCR provide better detection rates for the target species than the metabarcoding. I consider this study provide important information and knowledge for ocean ecology. However, the one analysis to estimate “corrected” read number is bit confusing (L492-516). In addition, some explanation is insufficient and there are several typos in this MS. I added detailed comments below.*

> Thank you very much for your comment and suggestion. We are sorry that we didn’t make it clear of explaining the MiFish read number adjustment and some other points. This time we added explanation about how we did the “quantifying adjustment of MiFish reads result with qPCR data” in the Materials and methods. We also added some other explanations (such as the explanation of water filtering method and the explanation of eDNA purification method) according to your suggestion. We deeply apologize for the typos, this time we made it more carefully in checking the MS to clean up the typographical errors.

*Detailed comments*

*L26: correct “invasively” to “noninvasively”.*

>We deeply apologize for the typo, we have changed the “invasively” to “noninvasively” (L26, line number is based on 'Revised Manuscript with Track Changes' file at "Hidden revisions" status for here and below).

*L52-54: Correct to “~the most reliable method. However, the ability of fish influences the catch efficiency and this method requires enormous amount of time in the open ocean, these factors making difficult to obtain high resolution data.”*

> Thank you very much for your advice. Wave changed the sentence based on your advice: “~the most reliable method. However, the escaping ability of fish influences the catch efficiency, and this method requires an enormous amount of time in the open ocean, which makes it difficult to obtain high resolution data.” (L50-53).

*L61-62: Is this due to open ocean? Reading this paper [11], it seems to be rather due to difficulty in detecting eDNA from Cetaceans. Other studies (e.g., Garcia-Vazquez et al. 2021) on open oceans are more suitable to refer to here.*

> Thank you for the advice. As you mentioned it seems that the failure of detecting killer whale here may be due to the difficulty in detecting eDNA from Cetaceans. Therefore, we have changed the [Róisín Pinfield](https://onlinelibrary.wiley.com/action/doSearch?ContribAuthorRaw=Pinfield%2C+R%C3%B3is%C3%ADn) et al. 2019 to the Garcia-Vazquez et al. 2021 which showed the difficulty of for detecting planktons in open ocean compared with coastal area for the reference [11]. We also changed the “fish and marine mammals” to “fish and zooplankton” (L59) and changed “in one study, although water samples were collected very near offshore killer whales *(Orcinus orca*), killer whale eDNA was not detected” to “For example, zooplankton were detected in the coastal region, but not in the open waters, in 1.5 L water samples using COI metabarcoding” (L60-61).

(Garcia-Vazquez et al. 2021, doi: 10.1038/s41598-021-86731-z.)

*L72: “require only a small volume of sample (<10μL). I think this is a minimum volume rather than standard for species specific PCR. You should describe a standard volume of qPCR samples and it is smaller than metabarcoding.*

> Thank you for your suggestion. Standard volume required for one qPCR reaction is about 2-2.5 μL (Minegishi et al. 2019, Takahara et al. 2013) and standard volume required for one reaction in metabarcoding varies among different studies (2μL in Markus et al. 2018, 10μL in Nathan et al. 2015). As sometimes required volume of metabarcoding is as small as qPCR, we decided to delete the sentence “require only a small volume of sample (<10μL)” when explaining advantages of qPCR (L71).

(Minegishi et al. 2019, doi: 10.1371/journal.pone.0222052.

Takahara et al. 2013, doi: [10.1371/journal.pone.0056584](https://doi.org/10.1371/journal.pone.0056584).

Markus et al. 2018, doi: 10.1038/s41598-018-23052-8.

Nathan et al. 2015, doi: 10.1111/1755-0998.12433.)

*L86-88: Why does this sentence appear in the paragraph of metabarcoding?*

*This does not fit here.*

> Sorry for causing the misunderstanding here. In this part, we wanted to introduce the disadvantage of both qPCR and metabarcoding here. To make it clear, we moved the introduction of MiFish (“For fish species, a new eDNA metabarcoding … in the eastern equatorial Pacific [19, 21].”) to the previous paragraph that introduced the qPCR and eDNA metabarcoding and their advantages. Then, we changed the remaining part in this paragraph to “However, both qPCR and eDNA metabarcoding face observational biases due to the degradation of eDNA, advection by ocean currents, and the inhibition of DNA amplification by additional substances [22-24]. In addition, amplification bias of high-throughput sequencing (HTS) can influence the sensitivity of eDNA metabarcoding [25-28]. High abundance of eDNA may inhibit the amplification of low-abundance eDNA through the competition of binding with metabarcoding primers [25-27]. Both issues can result in the loss of some species in eDNA metabarcoding analysis.” to introduce the disadvantage of both qPCR and metabarcoding here (L81-86).

*L92: Typo. Correct ”species□specific PCR” to “species-specific PCR.”.*

>Sorry for the special character, we have corrected it (L90-91).

*L94: Typo. Correct to “However, research comparison….”*

>Sorry for our mistake, we have corrected it (L92).

*L138: Typo. Correct to “An approximately…”*

>Sorry for our mistake, we have corrected it (L136). And we are very sorry that we made another mistake here. In fact, we filter approximately 7L water for each sample in this study, so we finally corrected here as “Approximately 7 L…” (L136).

*L138: How did you filter 10L of water using a Sterivex? Using aspirator?*

*You should describe the details of procedure.*

> Thank you for your suggestion. Now we added the details to show the procedure of water filtering here (L137-145).

“To perform filtering, inlet end of the Sterivex-GP pressure filter unit was attached to the 1/4 inch HB to M Luer lock (XX3002564; Merck Biopharma Co., Ltd., Tokyo, Japan), which was assembled into one end of a peroxide-cured silicon pump tube (L/S25, 96400-25; Yamato Scientific Co., Ltd., Tokyo, Japan). The pump tube was then fixed by tube cartridge (07519-70; Yamato Scientific Co., Ltd.) to the multi-channel pump head (07519-06; Yamato Scientific Co., Ltd.). The pump head was assembled into a digital pump (07528-10; Yamato Scientific Co., Ltd.). Finally, through the peroxide treatment silicon pump tube, the digital pump (rotation speed set to 60 rpm) pushed the water sample through the Sterivex-GP pressure filter unit from the plastic bag.”

*L155: You should briefly explain the eDNA extraction procedure.*

> Thank you for your suggestion. Now we added some explanation of eDNA extraction and purification here (L163-170).

“DNA extraction was performed using Charge Switch Forensic DNA Purification Kit (Thermo Fisher Scientific). Briefly, after removing RNAlater, 2 mL lysis mix (containing Lysis Buffer and 20 µL Proteinase K) was added to each Sterivex filter unit. The filter units were incubated at 55℃ for 30 min. Supernatant from each filter unit was collected in a new microcentrifuge tube. ChargeSwitch® Magnetic Beads (Thermo Fisher Scientific) were added to bind to the DNA. After washing the magnetic beads with Wash Buffer, DNA combined with Magnetic Beads was eluted with 150 µL of Elution Buffer for each sample and then transferred to a new microcentrifuge tube.”

*L167: Correct to “the average number of raw and clean reads was 600,718 and 138,476, respectively.”*

>Sorry for our mistake, we have corrected it (L177-178).

*L172: Don’t refer to an unpublished material.*

>As this article is already published now, we included it in the references list as [36] this time (L181).

36. Marty Kwok-Shing Wong, Shigenori Nobata, Shin-ichi Ito, Susumu Hyodo. Development of species-specific multiplex real-time PCR assays for tracing the small pelagic fishes of North Pacific with environmental DNA. Environ DNA. 2022 January 14. doi: 10.1002/edn3.275.

*L191: Add the interpretation of value of Pearon’s Phi-coefficient (e.g. 0.5 or higher means strong positive relationship, 0.3-0.5 means …, 0.1-0.3 mean… )..*

>Thank you for your suggestion. We added some interpretation of Phi-coefficient value. However, as precise meaning of coefficient value depends on the freedom of data, we more depended on the p-value (Fisher's exact test) here rather than using “crude estimates” coefficients meaning list. (L211-214)

“The value of the Pearson’s Phi-coefficient ranges from -1 to +1, where +1 (-1) indicates perfect agreement (disagreement) and 0 indicates no relationship. As the precise meaning of the coefficients (strengths of relationships) depends on the freedom of data, we also calculated the p-value of Fisher's exact test.”

*L196:Typo. Delete “We”.*

>Sorry for the typo, we have corrected it (L197).

*L218-220: Add the information regarding body size of the target species base on net sampling.*

>Thank you for the suggestion. We added the information of the body size of the target species collected by the net sampling here (L265-268).

“The target fish species caught in the net comprised 158 sardine (40.85-66.80 mm body length, 0.85-3.74 g body weight; 0.246 kg in total), 289 chub/blue mackerel (16.99-64.08 mm fork length. 0.04-2.75 g in body weight; 0.044 kg in total), and 40 anchovies (34.99-68.68 mm body length, 0.35-3.24 g in body weight; 0.044 kg in total).”

*Fig.3 is not necessary because this information is described in Table1.*

>Thank you for your advice. As you said, the information of Fig 3 is also included in the Table 1. However, when comparing the detection rate from qPCR and from MiFish among five target fish, we think the figure version will be clearer to understand. So, here we kept the Fig 3 (L300) and to prevent the information repetition, we moved the original Table 1 to the Supporting information part as S7 Table.

*L274-: You mentioned that* *Mifish metabarcoding failed for* *63 samples. Even so, did you use the results of all the samples for this analysis?*

>Thank you for your question. By your question, we realized that we made a mistake. We did not include the 63 samples that failed in this Phi-coefficient examination, thus we corrected “247 OceanDNA samples” to “184 OceanDNA samples” here (L312).

*L274-279: As above mentioned, I don’t know how large the value of Phi-coefficient means a strong relationship between two variables. Please explain this in the statistical analysis section..*

>As our answers for your question to original L191, we have added some interpretation of Phi-coefficient value, but we more depended on the p-value (Fisher's exact test) here. From the p-value we can see statistically significant positive correlations were found for chub/blue mackerel, sardine, and saury as well as for pooled data (L316-320).

*L299-322 and Fig.5: It is difficult to distinguish the results from MiFish and qPCR in Fig 5 because both marks are small and overlayed. Enlarge the mark of each mark and add a graph legend for them.*

>Thank you for suggestion. We divided the original Fig 5 into two figures, Figs 5 and 6, for PLOS limited the max size of one single figure. Now the figures and the marks are larger, and we also added the legend of qPCR and MiFish. We hope the figures now are enough for reading the detection results from MiFish and qPCR (L342-354).

*L386-400: Although this analysis is important and valuable for discussion, this paragraph describes the analysis procedure and its results, which should be moved to “Materials and method” and “Results”.*

>Thank you for suggestion. We put the detailed procedure in the “Materials and method” (L221-230).

“To find the possible reasons for the different detection rate of the qPCR and MiFish methods, we tested two hypotheses. One hypothesis posited that the detection limit of the qPCR method is lower than that of the MiFish method. The other hypothesis posited that OceanDNA amplification of target species is inhibited by OceanDNA amplification of non-target species (amplification bias) in the MiFish method. To test whether the two methods have different DNA quantity detection limits, we divided the samples in which qPCR detected the target species into different groups according to the OceanDNA quantity of target species measured by qPCR and then compared the MiFish detection loss rate among all groups. The MiFish detection loss rate was defined as the percentage of samples in which MiFish failed to detect target species.”

And then, as you suggested, we built a part as “Influences of detection limit and amplification bias on detection rate” inside “Results” part to include the analyzing results (L370-387). Besides, we did some slight modification in the “Discussion” (L465-471).

*L419: Typo: “hada”?*

>Sorry for our mistake, we have corrected “hada tended” to “tended to be” (L479).

*L416-427 and 444-452: These sentences should be also moved to “Materials and method” and “Results”.*

>Thank you for your comment. We moved the explanation of methods in the “Materials and method” (L230-237).

“~To test the influence of the amplification bias in the MiFish method, we divided the samples in which qPCR detected the target species into the group in which target fish was not detected by MiFish (MiFish-0/qPCR-1 samples) and the group in which MiFish also detected the target fish (MiFish-1/qPCR-1 samples). We then compared the number of non-target OTUs and the chlorophyll-a concentration between these two groups using the Mann-Whitney U test to test the statistical significance. Non-target OTUs were OTUs that belonged to the non-target fish. Chlorophyll-a concentration was included for comparison because it represents the algae quantity, and algae DNA could not be amplified using the MiFish method.”

As for the analysis results in original L416-427, as we explained in the responses to the L386-400 question, we built a part inside “Results” to include them (L387-400). We also did some slight modification in the “Discussion” (such as in L480-486).

As for original L444-452, like we introduced in the “Materials and method” (S1 Text) the library preparation is the first step of the MiFish procedure. So, the MiFish method cannot be applied to samples in which the library preparation method failed. Till now, the reason of library preparation failure is undermined. Therefore, we thought it is better not to include it in “Results” part. However, library preparation failure did influence the detection ability of MiFish. Then, we included this part in “Discussion” to remind other researchers of this unsolved problem in MiFish method.

*L484: Typo. Correct to ”In the present study”.*

>Sorry for the typo. We have modified this sentence. (L532-535)

*L491: Is it really difficult to apply qSeq to Ocean DNA study? Have you ever confirmed? Are there any references?*

>Thank you for the question. When searching for the previous researches on fish metabarcoding, we didn’t find the record of using qSeq in open ocean eDNA samples. As you mentioned latter, several researchers succeeded performed qSeq for marine fish eDNA, but what they have in common is their samples were collected in coastal area, such as in the bay. For example, in Ushio et al. 2018, their marine eDNA samples were collected in coastal marine ecosystem in Maizuru Bay. We could not find any qSeq studies on open ocean fish. Therefore, we considered that it may be difficult to apply qSeq to OceanDNA when studying on fish (which was collected far away from the land, just as the Kuroshio Extension where we collected our samples). However, there is one research that performed qSeq in open ocean successfully for microorganisms (Lin et al. 2019), so we agreed that our original statement here is not proper enough.

To make the explanation proper and clear, we modified it as follows (L535-540).

“qSeq has also been used in eDNA studies on marine fish, but these studies were performed in environments close to the coast or in the bay [54]. For samples collected in open ocean far away from the land, such as the samples in this study, which were collected in the Kuroshio Extension, qSeq application may be difficult for fish study. Indeed, we could not find any previous study on fish with qSeq in the open ocean, although there has been a successful attempt with the microbiome community [55].”

(Ushio et al. 2018, doi: 10.3897/mbmg.2.23297.

Lin et al. 2019, doi: 10.1128/AEM.02634-18.)

*L496: Fig 9 should be corrected to Fig 8?*

>Thank you for the reminding. Sorry that we made a mistake in citing figure here. And because of the manuscript modification, now Fig 9 here is correct (L545).

*L519-520: Grammar error. Correct this sentence.*

>Thank you for your comment. Now we made it clear as follows (L567-569).

“MiFish read and qPCR quantity in this figure used the data of chub/blue mackerel (*Scomber japonicus* and *Sc. australasicus*) in the B-line, chub/blue mackerel in the C-line, anchovy (*Engraulis japonicus*) in the B-line, anchovy in the C-line, sardine (*Sardinops melanostictus*) in the B-line, and sardine in the C-line.”

*L487-489: Quantitative MiSeq sequencing (qMiSeq)has been also utilized in marine environments to monitor eDNA concentrations of multiple species simultaneously (Ushio et al. 2018 Metabarcoding and Metagenomics; Ushio 2019 Methods in Ecology and Evolution; Sato et al. 2021 Scientific reports).*

> Thank you very much for your supplementary introduction. As you mentioned, there are successful usage of Quantitative MiSeq sequencing (qMiSeq) in marine eDNA study. However, as mentioned above, rather than in the open ocean area away from the land, their sampling works are all performed in the areas close to the land, like in the coastal marine ecosystem (Ushio et al. 2018 and 2019) or around artificial reefs in Tateyama Bay (Sato et al. 2021). In fact, we also agree with the worth of trying qMiSeq in open ocean, but it may be difficult. So, we showed another possible way, to combine the use of qPCR and MiFish methods to achieve quantitative sequencing. To make it clear, we modified the text (L536-541).

*L492-516: I do not understand this calculation from your sentences. Why the relationship in Fig. 8 can be true for other samples? MiFish read percentages in this figure may also have amplification bias. In that case, you cannot use this relationship to estimate the correct fish composition. At least, you should use the equation to explain this calculation for easier understanding to readers. In addition, these sentences also include “Materials and method” and “Results” as above mentioned. Distinguish them from “Discussion”.*

>Thank you for the question and advice. To make the statement clearer, we added the explanation how we did the quantifying adjustment of MiFish reads result with qPCR data as follows in the “Materials and method” (L238-256).

“Considering the ability of MiFish to detect large numbers of species and the quantitative nature of qPCR, we attempted to combine the two methods to simultaneously determine the quantitative distribution of small pelagic fish species within fish community structures. We wanted to convert the OceanDNA read percentages detected by MiFish to OceanDNA quantities as an average view of the observation vertical sections. First, we investigated whether the MiFish read percentage (y) of the target species and qPCR quantity (x) showed a positive correlation. We used the data of sardine, anchovy, and chub/blue mackerel in the B- and C-line, since their detection rates were high (see Results). The MiFish read percentage indicates the selected species read percentage to the total OTU reads of all B-or C-line samples. Linear regressions were performed using statsmodels.regression.linear_model.OLS of Python. If a significant positive linear correlation between the MiFish read percentage (y) and qPCR quantity (x) was found, the adjusted DNA quantity of species n of all selected samples, Σ(*q_n_*), was calculated as:

$\Sigma{(q}_{n})=\Sigma[y_{n}*(q_{sardine}+q_{anchovy}+q_{mackerel})/(y_{sardine}+y_{anchovy}+y_{mackerel})]$,

where *q_sardine_*, *q_anchovy_*, and *q_mackerel_* are the qPCR quantities for sardine, anchovy, and chub/ blue mackerel, respectively; and *y_sardine_*, *y_anchovy_*, and *y_mackerel_* are the MiFish read percentages of sardine, anchovy, and chub/ blue mackerel, respectively. The selected sample was one in which at least one of the sardine, anchovy and chub/blue mackerel was detected by both the MiFish and qPCR methods. The fish species composition was calculated using Σ*q_n_* and compared with the original composition.”

In our samples, we found a significant positive linear correlation between the MiFish read percentage and the qPCR DNA quantity (L548). Using the method above, we calculated the adjusted DNA quantity from MiFish read number of each fish species. We apologize for causing the misunderstanding, but the method here was not for solving amplification bias problem. What we tried to do is estimating the DNA quantity from MiFish read number.

As you pointed out, the linear MiFish-qPCR correlation we got here was based on data in this study, so may not apply to other eDNA studies. In fact, we did not intend that we discovered a universal quantitative relationship between qPCR and MiFish data. What we wanted to indicate is a possible way of combining usage of MiFish and qPCR. Other researchers can simultaneously quantify and sequence multiple fish species if they discover the MiFish-qPCR quantitative relationship in their own OceanDNA samples.

In this study, we studied about the validity of each eDNA analysis methods (represented by MiFish and qPCR) to help developing eDNA method for fish distribution survey in open ocean. After a series of study, we confirmed the validity of both methods but also recognized the lower detection rate of MiFish method. Although we also tested some possible reasons for lower detection rate of MiFish, our results were still not enough for overcoming this problem. On the other hand, the MiFish and other metabarcoding methods have the irreplaceable capability in multi-species analysis. Thus, it is difficult to choose one method as the “best” eDNA analysis method. So, in the final part of “Discussion”, we wanted to introduce this new idea, combining the usage of qPCR and MiFish, to show a new possible way for open ocean fish survey study in the future. Thus, we wished to keep this part in the position like in the original manuscript. But, to make our purpose clearer, we also did some modification (L530-532, 562-564).

*Reviewer #2: "Environmental DNA (eDNA) is increasingly used to invasively monitor aquatic animals in 27 freshwater and coastal areas." The word "invasively" in the first line of the abstract is not correct.*

*eDNA is a non-invasive technique compare to the traditional invasive technique. So application of this world here may cause misunderstanding. I recommend to replace this (invasively) with more relative and meaningful world.*

>Thank you for the comment. We are sorry for the typo here. We have replaced it with “noninvasively” (L26).
