# Supplementary material for: Comparison of species-specific qPCR and metabarcoding methods to detect small pelagic fish distribution from open ocean environmental DNA
Source: PLoS One. 2022 Sep 7;17(9):e0273670. doi: 10.1371/journal.pone.0273670 (PMC9451083; doi:10.1371/journal.pone.0273670)
Supplement: Attachment — Submitted filename: Response_to_Reviewers_YUzeshu _ 20220702.docx [file pone.0273670.s014.docx]

**Response to academic editor and reviewers**

We would like to thank the academic editor and reviewers again for their encouragement and efforts to review the manuscript. We believed we have done our best to address the comments below, including the methodology question raised by reviewer. We much appreciate the reviewers for your thorough and constructive reviews, which we think have greatly improved the manuscript. Our responses are in blue and red (red color marked what we modified in the manuscript), while the original comments from editor and reviewers are in black and italicized.

Response to reviewers

*Reviewer #1:*

*General comments to authors*

*Authors* *addressed the reviewer’s* *comments and revised the manuscript, which got largely improved. Meanwhile, I noticed that this paper does not contain information about negative controls of eDNA samples (i.e., filtering of distilled water or Milli-Q). Because sampling negative controls is important to check possible contamination during field and laboratory processes, authors should add sentences about it. Besides, I do not consider that the combining method of metabarcoding and qPCR results of the target species can provide reliable estimates of eDNA quantity of the non-target species (L238-256, L529-564 and 603-605). Therefore, I recommend authors to delete the related sentences and*

*figures about this method. I provided detailed comments about these points, as well as minor comments below.*

>Thank you very much for your comments. We are very sorry for our omission of negative control sample here. In the later cruises we collected the negative controls, and the MiFish/qPCR results showed no sign of contamination. We gave the detailed explanation about the problem of negative control below. As for the combining method of metabarcoding and qPCR, we agreed that it is difficult to be confirmed based on the present data, so we deleted the sentences and figures about it (we keep only one figure to illustrate our ideas for the future usage of both methods). And we also addressed other points you raised as showed below.

*Detailed comments*

*L62 The sentence of “The foregoing indicates that~” should be included*

*in the previous paragraph.*

> Sorry for our mistake, we have corrected it (L61).

*L108 Please add information of “scarce eDNA in open ocean” as “~ in the open ocean,* *where the concentration of fish eDNAs are expected to be scarce,* *has been insufficient.”*

*This will increase a necessity for this study.*

> Thank you very much for your advice, so now we wrote it as “~ in the open ocean, where the concentration of fish eDNA is expected to be scarce, has been insufficient.” (L107-108).

*L131 Correct to “~were collected from water depths of 5 to 300 m”.*

> Sorry for our mistake, we have corrected it (L132).

*L131-177 I noticed that this paper does not mention about negative controls of eDNA samples (i.e., filtering of distilled water or Milli-Q). Sampling negative controls is necessary to check possible contamination during field and laboratory processes. Please add sentences about it.*

> Thank you very much for pointing out the issue. We developed our water sampling protocols by learning from the previous eDNA studies on marine microorganisms. In those studies, the negative control is not common (like in Stoeck et al. 2010; Behnke et al. 2006). So, our sampling in this study also did not include negative control. However, as you pointed out, we also recognized the importance of sampling negative control in eDNA studies from the later studies. And we performed the negative control sampling in the later cruises (the data from which has not been submitted yet). We performed both qPCR and MiFish methods to the negative controls and we found no sign of contamination. As we used the same sea water sampling protocol in the later cruises, we also speculated that there is also no contamination in the KS-18-5 sampling.

Stoeck et al. 2010, <https://doi.org/10.1111/j.1365-294X.2009.04480.x>

Behnke et al. 2006, <https://doi.org/10.1128/AEM.72.5.3626-3636.2006>

We have added the explanation about the negative control problem in Discussion as follows (L402-413).

“Before discussing about the comparison between the characteristics of qPCR and MiFish methods, we must discuss about data quality of this study. Our seawater sampling protocol was relatively old and it was designed based on previous eDNA studies on the marine microorganisms, in which negative controls were not common [39, 40]. Thus, the KS-18-5 samples did not contain the negative controls. However, the later studies have shown the importance of negative controls to monitor possible contamination during the sampling [41, 42]. Therefore, in the cruises after KS-18-5, negative controls by filtering the Milli-Q water during water sampling for OceanDNA were performed. From the negative controls of the seven cruises after KS-18-15, no target fish DNA have been detected by the qPCR method and no reads have been detected by the MiFish methods (in the form of “library preparation failed as no PCR products obtained”). Although this cannot justify the lack of negative control in KS-18-15, the use of the same seawater sampling method suggested that there could be no contamination in the KS-18-5 sampling.”

*L229-232 You should decide this detection threshold of metabarcoding based on the result of negative controls (Yamamoto* *et al. 2016; Sato et al. 2021). At least, you should show read number of negative controls.*

> As we explained above, although we did not have negative control for KS-18-5, in the negative control we collected in later cruises, no read number or qPCR quantity was detected so we speculated there was also no contamination in KS-18-5. Even when contamination was not found, a threshold to avoid false presence is still needed for MiFish (just like in Sato et al. 2021). In our study, we set the threshold as 1% total read in each sample, which is similar but higher than in some previous studies (Li et al. 2019; Hänfling et al. 2016). In our study, the number (sum of all samples) of target OTUs meeting the 1% threshold is 149 and if we halve the threshold (to 0.5%), the number of target OTUs meeting threshold is 158, which only increased slightly. So, we speculated the 1% threshold is not very likely to cause serious change in our results.

Li et al. 2019, https://doi.org/10.1111/1365-2664.13352

Hänfling et al. 2016, <https://doi.org/10.1111/mec.13660>

(We also added these two articles into References to support our threshold setting. L200)

*L238-256 I understand your calculation for the adjusted DNA quantity of species n of all selected samples. However, I do not consider this calculation reliably estimate DNA quantity of the non-target species of qPCR because MiFish read percentage can vary with read number of other species as shown around 20 copies/μl of qPCR quantity in figure 9. MiFish read percentage can increase or decrease without change of its DNA quantity or read number if read numbers of other species change low or high, respectively. I consider such variability of MiFish read percentage can prevent from estimating reliable DNA quantity of non-target species. Therefore, I recommend authors delete the sentences*

*about this method and Figure 9-11.*

>Thank you very much for your advice. We analyzed and thought it again and as we felt it difficult to confirm the method confidently, so we deleted the sentences about this method and Figure 9, Figure 11. We added a new Fig 9 (modified from original Fig 10a) to illustrate our ideas about future usage of both methods.

*L262 Please add information about a depth range or depth layer (bottom or middle?) of net sampling in the main body and caption of Fig.2.”*

> Thank you very much for your advice. Now we modified the description of net sampling as “The net sampling data used were obtained by a pelagic trawling covered depth of 0-25 m that were performed during a cruise by the R/V Soyo-Maru vessel of the Japan Fisheries Research and Education Agency (net sampling station is shown in Fig 1), on May 11, 2018.”(L245-246), and we also added the information in caption of Fig.2 as “~net sampling (pelagic trawling, at depth of 0-25 m)” (L261). Although the net sampling only covered a narrow depth range, small pelagic fish are known for their diel vertical migration behavior, as showed in (Yasuda et al. 2018; Matsuo et al. 1997; Ohshimo 1996; Stenevik et al. 2007;), so we thought it is better to compare the net sampling data here with OceanDNA sampling data in a wider depth rage.

Yasuda et al. 2018, <https://doi.org/10.3354/meps12636>

Matsuo et al. 1997, <https://agris.fao.org/agris-search/search.do?recordID=JP1997005975>

Ohshimo 1996, <https://doi.org/10.2331/fishsci.62.344>

Stenevik et al. 2007, <https://doi.org/10.2989/AJMS.2007.29.1.12.77>

*L325 and Fig.4 Checking figure 4, explanation in the caption seems to be incorrect. Pooling-4 and pooling should correspond to (f) and (e), respectively.*

>We are very sorry for this mistake. As you said, the pooling-4 and pooling correspond to (f) and (e), respectively. And we have corrected it in the caption of Fig 4 (L308-310).

*L488 In the discussion, you should infer possible reasons for preparation failures in MiFish libraries.*

> Thank you very much for your advice, now we have added the discussion about the possible reasons for library preparation failures in MiFish as follows (L496-504).

“One critical step in the MiFish library preparation is the first-round PCR that the universal PCR primers amplify the target gene fragments across the target taxa [57]. Therefore, the library preparation failure is likely to happen in the samples with virtually no DNA of target taxa. Indeed, in the negative controls we collected during the later cruises, the library preparation also failed, as mentioned earlier. The lack of target taxa DNA is also possible in seawater samples for the high proportion of microbial DNA and the sparse distribution of fish eDNA [10]. Although MiFish primers were reported to be very effective [58, 59], there are still possible problems like PCR dropout which can lead to amplification failures of existed fish DNA in samples [57]. As shown in our study, practical library preparation of OceanDNA could be challenging in the MiFish method.”

*L492-494 How could you still calculate the detection number ratio with samples failed library preparation? Even if you failed library preparations, could you still get these results?*

> Sorry for causing the misunderstanding. As you said, if we failed the library preparation in a sample, the MiFish procedure cannot work (like we introduced in the Materials and methods), so target fish detection cannot be found in it. Here we introduced the detection number ratio when included the samples with failed library preparation because we wish to highlight its negative impact to MiFish and call attention from other researchers to deal with the problem of library preparation failure.

*L529-564 and 603-605. As mentioned above, I am not convinced that combining method of qPCR and MiFish can reliably estimate the adjusted quantity of DNA in the non-target species of qPCR because MiFish read percentage can vary with other species reads. I recommend authors to delete these sentences and Figure 9-11.*

> Thank you very much for your advice. As mentioned above, we deleted the sentences about the combining method and Figure 9, Figure 11. And we made a little modification of original L 509-528 and used it as the end part of discussion as “Perspectives for future development of OceanDNA analysis methods in marine fish distribution surveys” (L516-559), in which we emphasized the value of both qPCR and MiFish and suggested the further development as follows. Although we agreed the combined method is not reliable at the present, we added a new Fig 9, (modified from original Fig 10a) to illustrate our ideas that combining usage of both methods could be feasible and useful in the future.

“Given the information above, we propose combining the qPCR and MiFish methods in OceanDNA analysis to monitor quantitative distribution of small pelagic fish species with information of fish community structures. The qPCR method provides quantitative estimates and special distribution of target fish species as shown in Figs 5 and 6. On the other hand, using MiFish data of the water samples in which the qPCR method detected the target species (we called them selected samples), fish community coexisting with the target fish can be inferred. For example, we estimated the fish community coexisting with sardine and chub/blue mackerel (Fig 9). This calculation assumed that DNA quantity is proportional to the MiFish read number over the sum of the samples. At this stage, the MiFish data does not have quantitative information. Therefore, the ratios in Fig 9 do not represent relative abundance. To overcome this issue, quantitative sequencing (qSeq) technique, which adds a random sequence tag to the target sequence before PCR in the library preparation to estimate the DNA quantity from read number found in the metabarcoding [61, 62], is required. qSeq has been used in eDNA studies on marine fish, but these studies were performed in environments close to the coast or in the bay [63]. We could not find any previous study on fish with qSeq in the open ocean, although there has been a successful attempt with the microbiome community [64]. Even with qSeq, there is still a risk that the ‘read number-DNA quantity’ relationship varies among different species, which can be examined by qPCR data of several target fish. In addition, the added random sequence tag for qSeq consumed some reads in the sequencing, which may reduce the read number we can obtain from the target fish DNA. In our preliminary experiments (data has not been submitted), adding the sequence tag increased number of non-detected species in MiFish, which suggested the possible difficulty of qSeq in OceanDNA. Thus, we suggest the combination of the qPCR and MiFish methods as a possible way to detect a large number of species that coexist with the target fish. The combined methods will permit a comprehensive understanding of the quantitative distribution of small pelagic fish within fish community in the open waters.”

We also made a little modification in the Abstract and Conclusions and (L44-46, L587-588).

*Reviewer #2: (No Response)*
