# Supplementary material for: Comparison of species-specific qPCR and metabarcoding methods to detect small pelagic fish distribution from open ocean environmental DNA
Source: PLoS One. 2022 Sep 7;17(9):e0273670. doi: 10.1371/journal.pone.0273670 (PMC9451083; doi:10.1371/journal.pone.0273670)
Supplement: Attachment — Submitted filename: Response_to_Reviewers_YUzeshu 20220812.docx [file pone.0273670.s015.docx]

**Response to academic editor and reviewers**

We would like to thank the academic editor and reviewers again for their encouragement, and efforts and patience to review the manuscript. We revised our manuscript based on the new comments. Again, we really appreciate the reviewers for your thorough and constructive reviews, which we think have greatly improved the manuscript. Our responses are in blue and red (red color marked what we modified in the manuscript), while the original comments from editor and reviewers are in black and italicized. We also introduced the changes to our reference list below.

Response to reviewers

*Reviewer #1:*

*General comments to authors*

*Authors addressed the reviewer’s comments and improved the manuscript. I just provided minor comments below.*

> Thank you very much for your review and constructive comments. We revised our manuscript based on the comments you provided.

*Minor comments to authors*

*L62 Correct to “OceanDNA has the potential to be a valuable fish survey method”.*

> We are very sorry for the typo here, and we have corrected it (L62).

*L402-413 I understand your excuses regarding no negative controls in this study. In these sentences, both KS-18-5 and* *KS-18-15 appeared, and confusing. The KS-18-15 was mistakenly mentioned? Or such a cruise was also present?*

> We are very sorry for this mistake. The “KS-18-15” cruise was a typo of “KS-18-5”. We corrected this mistake (L408-412).

*L526 Correct to “three metabarcoding primers”.*

> Sorry for our mistake, we have corrected it (L525).

*L547-548 The qSeq is different from the qMiseq used in Ushio et al.(2018), so the reference of [63] is incorrect. The latter method uses internal standard DNAs to create sample-specific regression lines*

*between the numbers of sequence reads and DNA copies for estimating original DNA copy numbers.*

> We are very sorry for this mistake and thank you for pointing it out (and we realized [64] also used method different from the qSeq). We found the qSeq method has not been performed to marine fish yet, in the previous studies. Therefore, we removed the wrong references and sentence, and simplified the statement here as follows (L546-549).

“However, we could not find any previous study on fish with qSeq in the open ocean. Besides, even with qSeq, there is still a risk that the ‘read number-DNA quantity’ relationship varies among different species, which can be examined by qPCR data of several target fish”

Changes of the reference list:

1) Original 55. Jason B. Alexander, Michael Bunce, Nicole White, Shaun P. Wilkinson, Arne A. S. Adam, Tina Berry, et al. Development of a multi-assay approach for monitoring coral diversity using eDNA metabarcoding. Coral Reefs. 2019 November 22. doi: 10.1007/s00338-019-01875-9.

was changed to “55. Laura M. Gargan, Paul R. Brooks, Siobhan R. Vye, Joseph E. Ironside, Stuart R. Jenkins, Tasman P. Crowe, et al. The use of environmental DNA metabarcoding and quantitative PCR for molecular detection of marine invasive non-native species associated with artificial structures. Biol Invasions. 2021 November 09. doi: 10.1007/s10530-021-02672-8.”

2) Original 66. Anna M. McKee, Stephen F. Spear, Todd W. Pierson. The effect of dilution and the use of a post-extraction nucleic acid purification column on the accuracy, precision, and inhibition of environmental DNA samples. Biol Conserv. 2015 March. doi: 10.1016/j.biocon.2014.11.031.

was changed to “64. Cindy Bessey, Simon N. Jarman, Oliver Berry, Ylva S. Olsen, Michael Bunce, Tiffany Simpson, et al. Maximizing fish detection with eDNA metabarcoding. Environ DNA. 2020 February 20. doi: 10.1002/edn3.74.”

3) Original 63. Masayuki Ushio, Hiroaki Murakami, Reiji Masuda, Tetsuya Sado, Masaki Miya, Sho Sakurai, et al. Quantitative monitoring of multispecies fish environmental DNA using high-throughput sequencing. Metabarcoding Metagenomics. 2018 March 14. doi: 10.3897/mbmg.2.23297.

was deleted.

4) Original 64. Yajuan Lin, Scott Gifford, Hugh Ducklow, Oscar Schofield, Nicolas Cassar. Towards quantitative microbiome community profiling using internal standards. Appl Environ Microbiol. 2019 February 20. doi: 10.1128/AEM.02634-18.

was deleted.
